# Patient perspectives on accessing eye-related healthcare from rural communities

**DOI:** 10.1038/s41433-024-03266-z

**Published:** 2024-07-24

**Authors:** Prethy Rao, Rajeev Ramchandran, Kira Baldonado, Stephan Hüsler, Marina Sutter, Larissa S. Moniz, Mohamed Akrout, Allon Jacobs

**Affiliations:** 1 Adult & Pediatric Retina Specialist, Retina & Vitreous of Texas, Beaumont, TX USA; 2 Department of Ophthalmology, University of Rochester Medical Center, Rochester, NY USA; 3 Prevent Blindness US, Chicago, IL USA; 4 Retina Suisse, Zürich, Switzerland; 5 Retina NZ Inc., Palmerston North, New Zealand; 6 Fighting Blindness Canada, Toronto, ON Canada; 7 Hoffmann-La Roche, Basel, Switzerland; 8 Genetech Inc., South San Francisco, CA USA

**Keywords:** Health care, Health services

## Introduction

Rural populations frequently encounter substantial barriers to accessing ophthalmology care, often resulting in delayed diagnosis and less effective disease management [1, 2]. This patient- and provider-centred multinational study explored the challenges patients and caregivers encounter and proposed improvements for rural communities.

## Methods

**Fig. 1:**
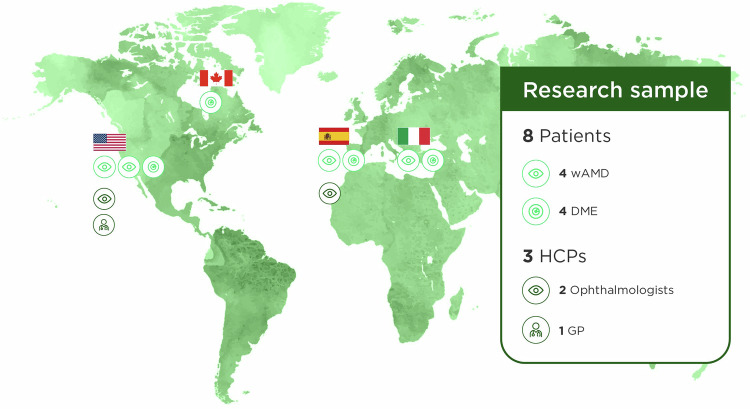
Patient and specialist demographics. DMO diabetic macular oedema, GP general practitioner, wAMD wet age-related macular degeneration.

A social media listening analysis and 60-min virtual discussions were conducted in Italy, Spain, Canada, and the United States with four patients with age-related macular degeneration and four patients with diabetic macular oedema, as well as with two ophthalmologists and one general practitioner (Fig. 1). Results were compiled and analysed to identify specific barriers and solutions in ophthalmology care for rural populations.

## Challenges identified

### Perceived challenges in accessing ophthalmology care

Among the many challenges associated with rural living identified by the study participants, the main burden for all patients was the need to travel extensive distances to attend specialised clinics (Table 1 – case studies), with half of the patients citing the lack of available public transport services as a major barrier. The main reasons patients are unable to drive themselves to their visits were due to impaired vision or a procedure such as injections or eye dilation. Therefore, they rely on family/friends for support and time-sensitive visits depend on caregiver availability. In addition, poor ophthalmology health outcomes are accentuated by socioeconomic factors. For instance, four patients reported their limited ability to work and the financial pressure their condition poses on them, while the physicians acknowledge that populations in rural areas tend to be financially disadvantaged, with many not having appropriate health insurance (particularly in the USA), compared with those living in urban areas [2–4].

**Table 1:** Selected case studies of patients and a healthcare professional relating to the treatment challenges and desired solutions for individuals living in rural areas.

	Patient with wAMD (USA)	Patient with DMO (Italy)	Ophthalmologist (Spain)
**Background**	• Diagnosed with wAMD ~2 years ago • Lives in a small town with access to a GP who is within a 1-h drive	• A 51-year-old living in a small town surrounded by mountains • Has had insulin-dependent diabetes for >20 years with a diagnosis of macular degeneration within the last 5 years	• Working in a tertiary urban hospital who also attends rural villages around the city ~10% of patients would fall in the definition of rural
**Travel/proximity to accessing specialised centres**	• Specialist centres are up to a 5 h drive away and there are no public transport options • Reliant on spouse for travel; however, they also have difficulties with driving, which can lead to missed appointments	• Travels every 4 months to their main specialist clinic and, in between, can attend a hospital that is closer for diabetes assessments and maculopathy management • Specialist centre is 30 km away, with no direct connections via public transport; however, a privately run local transport service does exist • The town’s GP is only available twice a week, but prescriptions are now managed online to help reduce the pressure	• Rural areas, especially the smaller ones, have limited means of transportation which are not available throughout the day • The difficulties accessing treatment centres means healthcare providers have to consider appropriate treatment options that will help treatment compliance and reduce potential absenteeism from follow-up appointments
**Treatment selection**	• Only received dietary/supplements advice • Has not received intravitreal injections as doctors felt they may not be suitable	• Receives intravitreal injections every 4–6 months • They cannot drive home following treatment and rely on family/friends to go to their treatment-administration appointments	• An early diagnosis reduces morbidity and prognosis, ultimately leading to better disease management • Provides intravitreal injections
**Additional pressures** **(financial/isolation etc.)**	• Medical centres try to accommodate travel needs but appointments can be delayed for 2 months if rescheduling is required • Dealing with local pharmacies can be frustrating due to treatments being unavailable, and the long wait times for prescriptions to be processed	• The patient is nervous about attending appointments and requiring additional treatment but is confident because they feel they are treated in an excellent way by the diabetologist, ophthalmologist, endocrinologist, and cardiologist	• The main impact on the patient is their dependency on others, as well as the infrequency of transportation
**Desired solutions**	• Virtual appointments would be extremely helpful, particularly if OCT devices could be used to perform examinations at home, to reduce the burden of travelling to see a healthcare professional • Special transport services to support getting to clinics would be of value and the patient would support with funding that option	• Transport solutions would help • Injections must be administered by the medical team at the clinic but for ad hoc questions it is useful to have access to a nurse for advice over the phone	• Would like patients to have specialised care available closer to them for easier access, with specialist doctors not just located in primary healthcare centres • The clinic tries to organise tests and treatments for patients in a single day and the medical team work flexible shifts to try to accommodate patient needs • Being able to provide intravitreal injections at treatment centres closer to the patient would have a positive impact • A dedicated helpline for any queries regarding symptoms, treatment or travel issues would provide emotional support which is much appreciated by patients • Implementing telemedicine for larger towns could help make managing patients easier with more convenient access to support • Diagnostic screening methods that can be implemented closer to the patient without specialist support could allow for more focused access to care

*DMO* diabetic macular oedema, *GP* general practitioner, *OCT* optical coherence tomography, *wAMD* wet age-related macular degeneration.

### Considerations from a provider perspective

Medical centres adapt to the patient’s needs by scheduling multiple appointments on the same day, potentially leading to extended working hours for healthcare professionals (HCPs). A USA-based ophthalmologist suggested HCPs occasionally choose treatments that require less frequent clinic visits (e.g., Panretinal Photocoagulation) to ease the challenges for patients, ultimately aiding treatment compliance. However, it was noted that considerations beyond the patient’s travel impacted their decision, including the cost and approval of treatment by insurers. Furthermore, concerns regarding local pharmacies not stocking specialist medication (e.g., eye drops) were raised, as this could delay treatment.

### Desired solutions

Caregiver/community groups, county-based or insurance-supported efficient healthcare transportation could reduce the burden for patients and their caregivers. Furthermore, digital health solutions, including virtual consultations and the use of digital devices (e.g., home-based Optical Coherence Tomography), could help reduce the cost of travel while providing medical support [5]. Lastly, selecting treatments associated with low administration frequency could potentially improve treatment adherence, disease management and ultimately the patient’s quality of life.

## Conclusions

This study highlights the barriers to patient care in the rural setting. Although measures exist to support patients in rural locations, resources are inconsistent and often limited. Improvements in healthcare transportation, coordination with local pharmacies, implementation of digital health solutions, and treatments with a lower frequency burden could help alleviate the current challenges.

## Data Availability

The data generated in this study are available within the article; additional information is available upon request from the corresponding author.
